# Correlation of nutrition-associated parameters with non-relapse mortality in allogeneic hematopoietic stem cell transplantation

**DOI:** 10.1007/s00277-021-04736-0

**Published:** 2021-12-21

**Authors:** Judith Schaffrath, Tanja Diederichs, Susanne Unverzagt, Maxi Wass, Ulrike Gläser, Thomas Weber, Mascha Binder, Carsten Müller-Tidow, Lutz P. Müller

**Affiliations:** 1 Department of Internal Medicine IV, Hematology and Oncology, Martin Luther University Halle-Wittenberg, Halle (Saale), Germany; 2 Institute of Nutrition, Consumption and Health, Faculty of Natural Science, University Paderborn, Paderborn, Germany; 3 Institute of General Practice and Family Medicine, Martin Luther University Halle-Wittenberg, Halle (Saale), Germany; 4 Department of Internal Medicine V, Hematology, Oncology and Rheumatology, University of Heidelberg, Heidelberg, Germany

**Keywords:** Allogeneic stem cell transplantation, Nutrition-associated parameters, BMI, Albumin

## Abstract

Outcome of allogeneic stem cell transplantation (alloSCT) is hampered by substantial non-relapse mortality (NRM). Given its impact on organ function and immune response, the nutritional status has been suggested as relevant for NRM. We aimed to evaluate the association of NRM with nutritional status prior to alloSCT and in the post-SCT course. In a retrospective single-center study, we analyzed 128 alloSCTs. Besides standard characteristics, nutrition-associated parameters BMI, serum total protein, and serum albumin were recorded before conditioning and at various time points after alloSCT. Association with NRM was evaluated by univariate and multivariate survival analysis. The cohort comprised patients with a median BMI of 26 kg/m^2^ (16.7–46.9 kg/m^2^), median serum total protein of 59 g/l (41–77 g/l), and serum albumin of 36 g/l (22–46 g/l) before SCT. NRM at *d*_+100_ was 14.8% and at 1 year 26.6%. Prior to SCT, only serum albumin deficiency was associated with increased NRM (*p* = .010) in multivariate analysis. After SCT (*d*_+30_ and *d*_+100_), all nutrition-associated parameters decreased (*p* < .002), but no association of deteriorating nutritional status with NRM was found. In multivariate analysis, serum albumin (*p* = .03) and severe albumin deficiency (*p* = .02) correlated with NRM at *d*_+30_ and *d*_+100_, while BMI and serum total protein did not. In our study, albumin deficiency, particularly prior to alloSCT, shows a strong correlation with NRM. This finding may add to monitoring, risk evaluation, and counseling of patients and serve as a rational for interventions to improve the nutritional status in patients undergoing SCT.

## Introduction

Allogeneic hematopoietic stem cell transplantation (alloSCT) represents a curative therapy for various malignant and non-malignant hematological diseases [1]. However, despite improvement, its success is still limited by its high toxicity. Around 20–32% of the patients die in consequence of non-relapse mortality (NRM) [2–4]. Predominant reasons are lethal infections, organ failure, and both acute and chronic graft-versus-host disease (GvHD) [5, 6]. Established risk factors for NRM include age [4, 7] and comorbidities [8] as well as conditioning intensity in older patients [9, 10]. The most common score used to assess the NRM risk is the HCT-CI score [11]. Despite the fact that alloSCT causes relevant metabolic changes [12] with an estimated increased basal metabolic rate of 130–150% [13] and that a deficient nutritional state is associated with worse outcome [14], the HCT-CI contains only one nutrition-related parameter which is obesity, i.e., a body mass index (BMI) of > 35 kg/m^2^. However, there is conflicting evidence from different trials on the impact of obesity on alloSCT outcomes and NRM risk, and therefore the relevance of the BMI is still disputed [15–19].

Given its impact on organ function and immune response, a number of studies have explored nutrition-associated parameters in the setting of alloSCT; however, little data exist on their exact role in NRM [20] and no verified and easily accessible parameter for nutritional status monitoring specifically in the alloSCT setting has been established.

Possible parameters include weight as well as BMI or its categories based on WHO standards [21, 22]. Regarding an association of BMI prior to SCT with outcome conflicting results have been reported. Various studies show reduced survival for patients with obesity [15], while other studies did not support this finding [16–18]. There is agreement on a general reduction of BMI post-SCT [23–26], but data on the relevance of BMI change in the post-SCT course have only scarcely been analyzed.

Other parameters related to the nutritional status are serum total protein and serum albumin. For both association with outcome has been reported for different hemato-oncological entities, e.g., peripheral T-/NK-cell lymphomas [27]. A prognostic relevance for SCT has been suggested, but is disputed [28, 29].

A better understanding of nutrition particularly in the post-SCT course and its relevance for NRM may not only aid in assessing prognosis but also in designing interventions to improve outcome. The benefit of such interventions has been discussed, but data remain unclear [30, 31].

Given the sparse data particularly on the relevance of post-SCT nutritional status, we evaluated the association of nutritional status assessed by BMI, serum total protein, and albumin at different time points prior and after alloSCT with NRM-related outcome.

## Patients and methods

### Patients

A total of 126 adult patients (age ≥ 18 years) who received a first and second (two cases) alloSCT at our center between January 2005 and September 2013 were included. The two patients with a second alloSCT were 11 and 22 months disease-free before relapse leading to their second alloSCT. As this interval was considered sufficient to reconstitute nutritional status, both patients were included and total cohort size was 128 alloSCTs.

Patients received an alloSCT from either an unrelated or related donor. In the absence of an HLA-matched sibling, the search for an unrelated adult donor was conducted in collaboration with the German National Bone Marrow Donor Registry (Zentrales Knochenmarkspender-Register Deutschland, ZKRD).

Nutritional support during alloSCT was performed according to local standards (in-house standard operating procedure) following the recommendations of the European Society of Clinical Nutrition and Metabolism (ESPEN) and the German Society for Nutritional Medicine (DGEM). During neutropenia < 500/µl, a low germ and moist steam sterilized light-balanced diet was offered and patients were encouraged to maintain a sufficient oral food intake for as long as possible. On the basis of DGEM recommendations, indications for initiation of parenteral nutrition were inability to ingest food orally for > 3 days, enteral calorie intake of < 500 kcal per day for > 5 days, > 3 days in patients with severe malnutrition, or < 60% of daily requirement for > 10 days, therapy refractory emesis, diarrhea, ileus, gastroparesis or high-grade mucositis, prolonged inappetence, and prevention of catabolic metabolism.

### Data collection and general SCT data

Data were collected retrospectively in our single-center study. Sources for data were inpatient and outpatient documentation. All patients had consented to treatment according to local standards. Data were handled pseudonymized during all stages of collection and analysis. Due to the pseudonymized analysis of retrospectively accrued data, no Institutional Review Board approval was deemed necessary. Patients consented to the collection and analysis of their data. Analyses were in line with the declaration of Helsinki.

Data were collected at the following time points: within 15 days prior to start of conditioning (preCond), the day of SCT ± 5 days (*d*_0_) and post-SCT on day 30 ± 5 days (*d*_+30_), day 100 ± 20 days (*d*_+100_), day 180 ± 10 days (*d*_+180_), and the last day of follow-up between day 110 and day 365 ± 10 days (*d*_+365_). Conditioning intensity was classified according to established criteria [32]. Patients were censored in case of relapse or disease progression or if treatment for another malignancy was started. Death after SCT without preceding censorship was regarded as non-relapse mortality (NRM). Varying *N* for different parameters at different time points resulted from censorship, preceding NRM and missing data.

### Analyzed nutrition-associated parameters

**Fig. 1:**
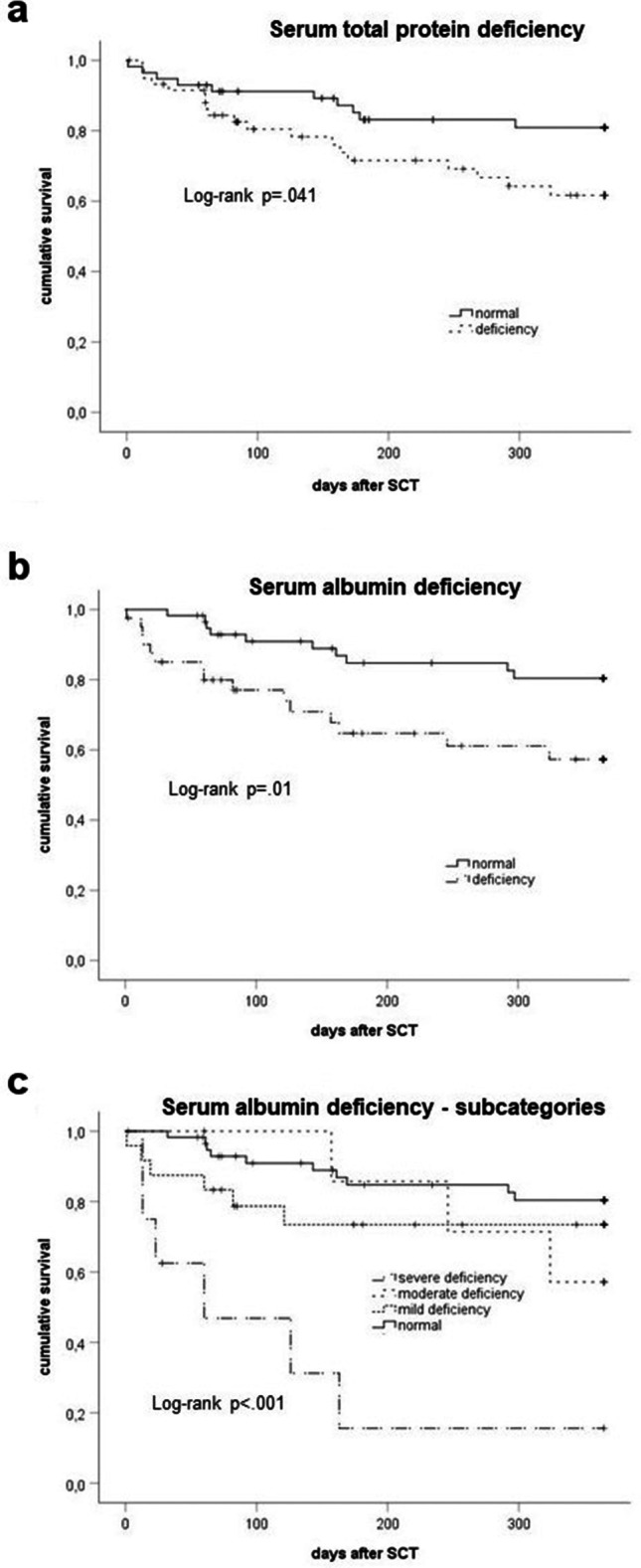
Analysis of survival (Kaplan–Meier) according to nutrition-associated parameter levels and distribution prior to alloSCT. **a** Serum total protein deficiency, **b** serum albumin deficiency, **c** categories of serum albumin deficiency; log rank testing according to Kaplan–Meier method

Patients’ body weight and height were derived from patient records. BMI (kg/m^2^) was calculated using body weight (in kg) divided by the square of the height (in m). For cross-sectional analyses, BMI was categorized according to WHO standards (BMI < 18.5 underweight, 18.5–24.9 normal weight, 25.0–29.9 overweight, ≥ 30.0 obesity). For analyses of longitudinal changes, an alteration in BMI of > 0.1 kg/m^2^ was classified as increase or of ≤ 0.1 kg/m^2^ as reduction. For serum albumin, a value of < 35 g/l was considered deficient with the following subcategories emerging as relevant during the analysis: mild deficiency 32–34.9 g/l, moderate deficiency 28–31.9 g/l, severe deficiency < 28 g/l, whereas serum albumin level ≥ 35 g/l was defined as normal. Subgrouping was initially based on current guidelines suggesting albumin levels < 32 g/l as a marker for cachexia [33]. Further analysis revealed a particular worse outcome for patients with albumin < 28 g/l (Fig. 1), and thus the present subgroups were analyzed. Regarding serum total protein, a level < 60 g/l was regarded as deficiency.

### Statistical analysis

All statistical analyses were conducted using software SPSS (version 25, IBM). Survival analysis was performed using the Kaplan–Meier method with log rank testing. Unifactorial associations of parameters with NRM were assessed using chi^2^-test. *T*-test for dependent variables was used when comparing parameters at different time points. Multivariate analysis was performed by binary logistic regression model with stepwise backward likelihood inclusion for parameters showing an association in univariate analysis and relevant cofactors as stated. Results are shown as odds ratio (OR) with 95% confidence interval (CI). All reported *p*-values are explorative.

## Results

### Patient characteristics and nutritional data at pre-SCT

General patient characteristics are summarized in Table 1. Upon start of conditioning patients had a median weight of 77.4 kg (minimum 46 kg, maximum 157 kg; *SD* 18.9 kg) and a median BMI of 26 kg/m^2^ (minimum 16.7 kg/m^2^, maximum 46.9 kg/m^2^, *SD* ± 5.2 kg/m^2^). The distribution of WHO-based BMI categories is shown in Table 1. Median serum total protein was 59 g/l (minimum 41 g/l, maximum 77 g/l; *SD* ± 7.1 g/l) and median serum albumin was 36 g/l (minimum 22 g/l, maximum 46 g/l; *SD* ± 4.7 g/l). This grouped patients according to normal or protein/albumin deficiency as shown in Table 1.

**Table 1:** Patient characteristics pre-SCT (*N* = 128)

Characteristic	Specificity	Values
Gender, N (%)	Female	50 (39.1)
	Male	78 (60.9)
Age, years		median: 56.4, range: 18–72
Karnofsky index, %		median: 90%, range: 40–100%
Diagnosis, N (%)	AML	58 (45.3)
	Lymphoma	35 (27.3)
	Myeloma	19 (14.8)
	Other ^1^	16 (12.5)
Disease status, N (%)	CR	56 (43.8)
	PR	34 (26.6)
	SD	28 (21.9)
	PD	8 (6.3)
	NA	2 (1.6)
Conditioning regimen, N (%)	MA	39 (30.5)
	RIC/NMA	89 (69.5)
Graft source, N (%)	Peripheral blood	124 (96.9)
	Bone marrow	4 (3.1)
Death, N (%)	Until *d*_+100_	23 (18.0)
	Cumulative until 1y	53 (41.4)
Cause of death, N (%) (cumulative until d_+100_ / until 1y)	Relapse	4 (3.1) / 19 (14.8)
	NRM	19 (14.8) / 34 (26.6)
	Lethal sepsis as cause of NRM	16 (12.5) / 31 (24.2)
Pre-SCT BMI, N (%)	Obesity	22 (17.2)
	Overweight	55 (43.0)
	Normal weight	46 (35.9)
	Underweight	5 (3.9)
Pre-SCT total protein, N (%)	Normal	57 (44.5)
	Deficiency	60 (46.9)
	NA	11 (8.6)
Pre-SCT albumin, N (%)	Normal	58 (58.6)
	Mild deficiency	24 (24.2)
	Moderate deficiency	9 (7.0)
	Severe deficiency	8 (6.3)
	NA	29 (22.7)

^1^Includes acute lymphatic leukemia, chronic myelogenous leukemia, osteomyelofibrosis, aplastic anemia; *AML*, acute myeloid leukemia; *MA*, myeloablative; *MM*, multiple myeloma; *NA*, unknown; *NMA*, non-myeloablative; *NRM*, non-relapse mortality; *CR*, complete remission; *PD*, progressive disease; *PR*, partial remission; *RIC*, reduced intensity conditioning; *SCT*, stem cell transplantation; *SD*, stable disease

As shown in Table 1, NRM at *d*_+100_ was 14.8% and at 1 year (1y) 26.6%. The main cause of NRM was infection with sepsis, accounting for 84.2% of NRM at *d*_+100_ and 91.1% of NRM at 1y.

### Association of pre-SCT nutritional status with post-SCT NRM

Survival analysis did not reveal an association of pre-SCT BMI with NRM and showed an increased NRM for patients with pre-SCT protein deficiency (Fig. 1a, log rank *p* = 0.041). Especially pre-SCT albumin deficiency was associated with worse NRM (Fig. 1b, log rank *p* = 0.010). Analysis for albumin deficiency subgroups revealed a particularly increased NRM for patients with pre-SCT severe albumin deficiency (Fig. 1c, log rank *p* = 0.001).

These results were confirmed by multivariate regression analysis for continuous levels of pre-SCT BMI, serum total protein, serum albumin and including covariates sex, age, and conditioning intensity. Only pre-SCT serum albumin but not BMI or serum total protein was independently associated with NRM as shown in Table 2 (stating the last step of the stepwise backward likelihood inclusion). Multivariate regression analysis stated a lower NRM with increasing pre-SCT serum albumin values (OR 0.82; 95%CI 0.73 to 0.92, *p* = 0.001). Furthermore, serum total protein deficiency and severe albumin deficiency were associated with clinically relevant increased NRM (OR 3.7; 95%CI 1.2–11.3 or OR 13.3; 95%CI 2.2–81.7) (Table 2).

**Table 2:** Results of multivariate analysis on association of nutrition-associated factors with NRM

Variable	*p*-value	OR [(Exp(B)]	95% CI
pre-SCT ^a^			
Serum albumin	.001	.82	.73-.92
Protein deficiency	.02	3.72	1.23–11.29
Severe albumin deficiency	.005	13.28	2.16–81.72
*d*_+30_^b^			
Serum albumin	.004	.80	.69–.93
Severe albumin deficiency	.005	6.30	1.75–22.71
*d*_+100_^b^			
Serum albumin	.03	.90	.81–.99
Severe albumin deficiency	.02	15.00	1.63–138.16

^a^Covariates at pre-SCT: age, sex, disease leading to SCT, conditioning intensity (MAC vs. others) and linear (BMI, albumin, protein) or categorized (BMI 4 categories, protein deficiency, 4 categories albumin) nutrition-associated parameters

^b^Covariates at *d*_+30_ and *d*_+100_: age, sex, conditioning intensity (MAC vs. others) and linear (BMI, albumin, protein) or categorized (BMI 4 categories, protein deficiency, 4 categories albumin) nutrition-associated parameters

### Longitudinal alterations of nutrition-associated parameters in the post-SCT course

**Fig. 2:**
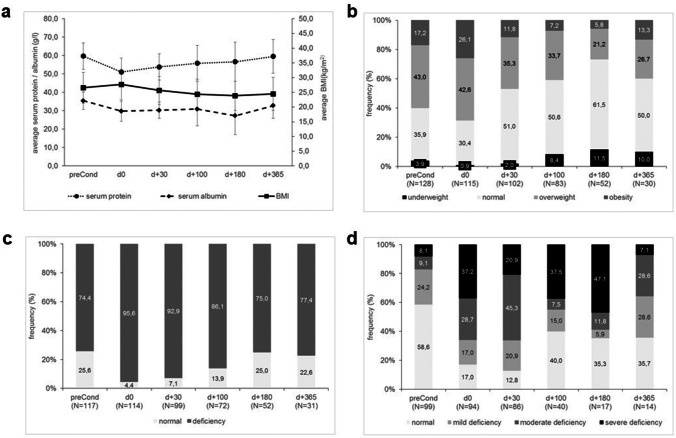
Distribution of nutrition-associated parameters in the course from prior to SCT (preCond) until 1 year post-SCT. **a** Mean BMI (right Y-axis), serum total protein and serum albumin (left Y-axis), error bars represent standard deviation, **b** BMI categories, **c** categories of serum total protein deficiency, **d** categories of serum albumin deficiency

Average levels of all parameters—BMI, serum total protein and serum albumin—decreased post-SCT (Fig. 2a). Given the expected alterations in fluid status and metabolism during conditioning further analysis was performed for levels at *d*_+30_ and *d*_+100_ but not at *d*_0_.

Compared to pre-SCT, on *d*_+30_ and *d*_+100_, a decrease in mean BMI (27.1 vs. 25.6 kg/m^2^ and 24,4 kg/m^2^, *p* < 0.0001 for both), mean serum total protein (60.4 g/l vs. 53.9 g/l and 55.8 g/l, *p* < 0.0001 for both), and mean serum albumin (35.9 g/l vs. 30.5 g/l and 31.7 g/l, *p* < 0.0001 and *p* = 0.001 respectively) was observed (*t*-test for dependent variables).

This corresponded to a relevant change (non-parametric testing for dependent variables) in the proportion of BMI subgroups and in the number of patients with a deficiency of serum total protein and serum albumin (for all *p* < 0.0001) on *d*_+30_ compared to pre-SCT (Fig. 2b–d). For *d*_+100_, a difference (non-parametric testing for dependent variables) was seen for the proportion of BMI subgroups (*p* < 0.0001) with a continuous increase of underweight and decrease of obese patients as well as in the number of patients with deficient serum albumin (*p* < 0.02). However, no difference was seen for the number of patients with serum total protein deficiency (*p* = 0.22).

### Association of NRM with longitudinal post-SCT alterations of nutrition-associated parameters

Given the evident changes in BMI status as well as in serum total protein and serum albumin on *d*_+30_ and *d*_+100_ (see Fig. 2), we analyzed whether the changes from pre-SCT status to *d*_+30_ and *d*_+100_ status correlated with NRM.

The occurrence of a decrease in serum total protein from pre-SCT to *d*_+30_ (log rank *p* = 0.81) and to d_+100_ (log rank *p* = 0.06) did not correlate with NRM (data not shown). Similarly, for serum albumin a decrease from pre-SCT to *d*_+30_ (log rank *p* = 0.39) and to *d*_+100_ (log rank *p* = 0.24) did not correlate with NRM-related survival (data not shown).

**Fig. 3:**
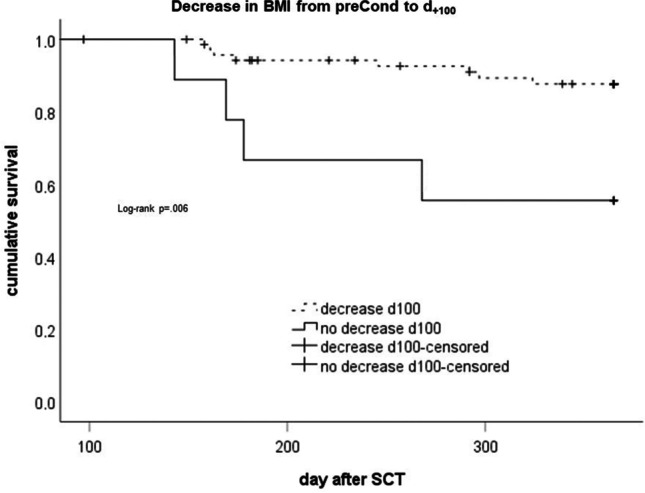
Analysis of survival (Kaplan–Meier) according to occurrence of a decrease in BMI from prior to SCT to *d*_+100_. Log rank testing according to Kaplan–Meier method

Interestingly, while a decrease in BMI from pre-SCT to *d*_+30_ did not correlate with NRM (log rank *p* = 0.24; data not shown), a decrease from pre-SCT to *d*_+100_ correlated with better NRM-related survival (log rank *p* = 0.006; Fig. 3), suggesting a better survival for patients with a decrease in BMI from pre-SCT to *d*_+100_ compared to patients with a stable or increasing BMI.

However, in multivariate regression analysis (binary logistic as well as Cox), BMI decrease from pre-SCT to *d*_+100_ showed no significant association (*p* > 0.1, data not shown) with NRM. Thus, it was not included in further multivariate analyses.

### Association of post-SCT nutrition-associated parameters with NRM

Given the altered nutrition-associated parameters in the post-SCT course, we analyzed NRM-related survival according to nutritional status on *d*_+30_ and *d*_+100_.

**Fig. 4:**
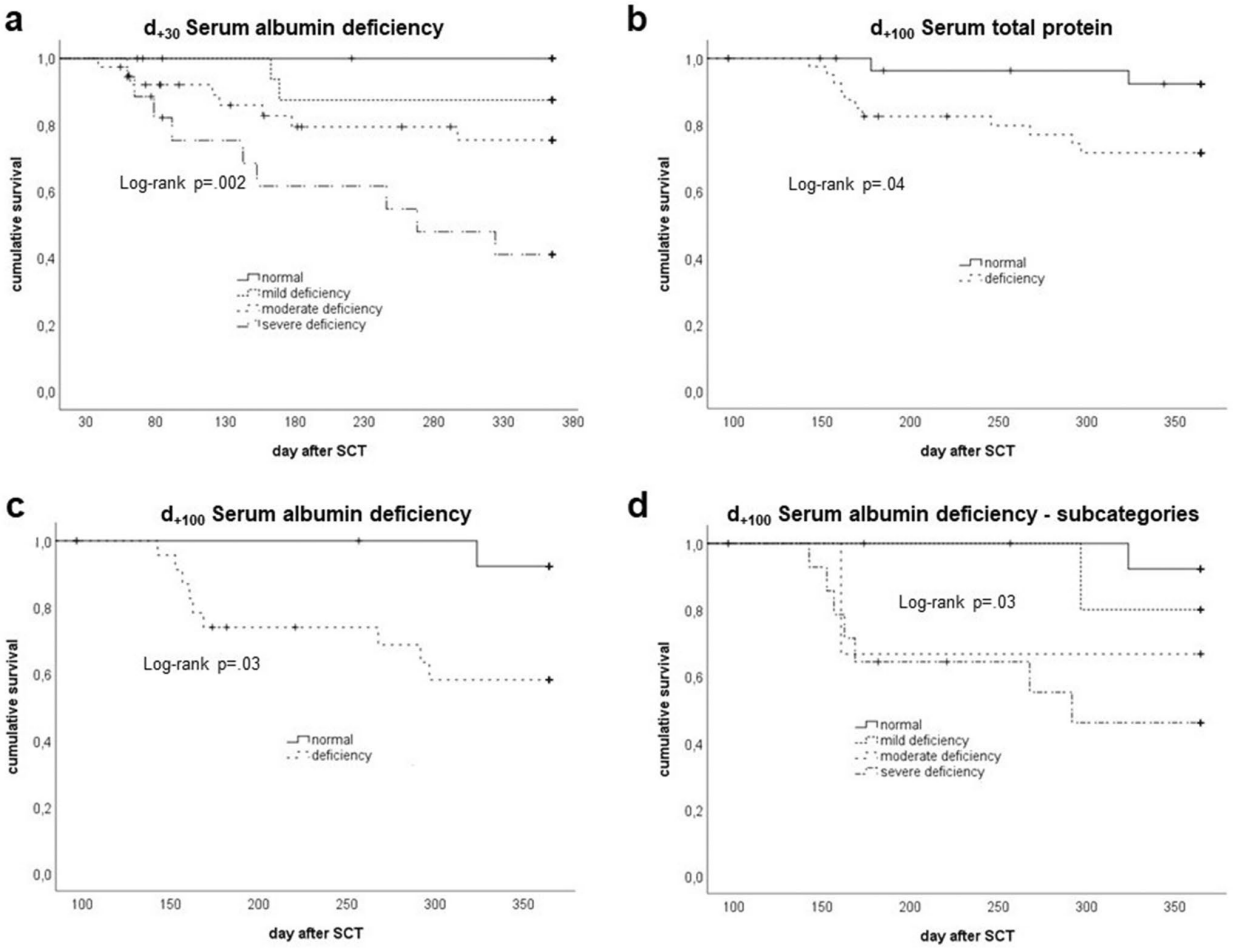
Analysis of survival (Kaplan–Meier) according to nutrition-associated parameter levels at the time post-alloSCT. **a** serum albumin levels at *d*_+30_ post-SCT, **b** serum total protein deficiency at *d*_+100_, **c** serum albumin deficiency at *d*_+100_, **d** subcategories of serum albumin deficiency at *d*_+100_; log rank testing according to Kaplan–Meier method

For *d*_+30_ status, according to the 4 BMI subgroups (log rank *p* = 0.75) as well as deficiency in serum total protein (log rank p = 0.11) and serum albumin (log rank p = 0.06) did not correlate with NRM (data not shown). However, severe serum albumin deficiency showed a strong correlation with very low NRM-related survival (log rank *p* = 0.002; Fig. 4a).

Similarly, for *d*_+100_, BMI subgroups (log rank *p* = 0.71) showed no correlation with NRM. However, patients with low serum total protein (log rank *p* = 0.04, Fig. 4b), low serum albumin (log rank *p* = 0.03; Fig. 4c), and particularly severe albumin deficiency (log rank *p* = 0.03; Fig. 4d) at *d*_+100_ had a low NRM-related survival.

As shown in Table 2, multivariate regression analysis confirmed a significant impact of serum albumin levels and of severe albumin deficiency on *d*_+30_ and *d*_+100_ on NRM. In contrast, neither BMI and BMI categories nor serum total protein or deficiency in serum total protein were associated with NRM at these time points.

## Discussion

Our present study belongs to the small number of studies analyzing nutrition-related data not only prior to alloSCT but also in the post-SCT course and in specific relation to NRM rather than overall survival (OS). In our single-center cohort, which comprised a typical distribution of donor, recipient, and transplant characteristics, we observed no association of BMI or its alterations in the post-SCT course with NRM. In contrast, serum albumin deficiency prior to SCT as well as at *d*_+30_ and *d*_+100_ post-SCT was consistently associated with NRM in multivariate analysis. While BMI, serum total protein, and serum albumin decreased in the post-SCT course, this decrease did not correlate with NRM.

Concerning the association between pre-SCT BMI and the outcome of allogeneic SCT, current data are inconsistent and comparability is hindered by different cut-offs of categorized BMI. Our finding of pre-SCT BMI being not associated with NRM is supported by other studies [16, 19], suggesting that neither underweight nor obesity prior to allogeneic SCT effect patients’ outcome. Due to the rising prevalence of adiposity, many studies focused on the impact of obesity on safety and efficacy of allogeneic SCT. In a retrospective analysis of CIBMTR data, Navarro et al. reported a comparable outcome of AML patients with a BMI ≥ 30 kg/m^2^, including OS [18]. These results were confirmed in obese (BMI ≥ 30 kg/m^2^) elderly patients (≥ 60 years) who underwent allogeneic SCT for myeloid malignancies and whose outcome including OS and progression-free survival were not affected by obesity [34].

Conversely, other authors reported an association of pre-SCT BMI with NRM-related survival [15, 17, 35, 36]. In a large cohort of 2503 adult patients, Doney et al. showed that both underweight (BMI ≤ 18.5 kg/m^2^) and very obese (BMI ≥ 35.0 kg/m^2^) patients had an increased NRM and underweight patients also had an elevated relapse and overall mortality rate [15]. In the retrospective analysis performed by Fuji et al. with registry data of 12,050 patients, NRM was significantly higher in the overweight and obese group (BMI ≥ 25.0 kg/m^2^) compared with the normal BMI group, mainly due to an increased GvHD- and infection-related NRM. However, this did not translate into a decreased overall survival for this patient group due to an increased risk of relapse in patients with a BMI ≤ 18.5 kg/m^2^ [35]. The study of Gleimer et al. showed the same association with a higher NRM in obese patients (BMI ≥ 30.0 kg/m^2^), yet no difference in OS due to a lower incidence of relapse in the obese patient group. NRM was mainly caused by acute and chronic GvHD, but despite a trend towards a higher incidence of both acute and chronic GvHD in obese patients, no difference compared to normal weight patients was shown [36]. Given the limited size of our cohort, we did not include GvHD as an additional covariate in our analysis.

The data reporting a higher NRM for obese patients are reflected in the addition of obesity defined by a BMI > 35 kg/m^2^ to the relevant comorbidities in the HCT-CI score, which is used for predicting transplant-related mortality risk prior to allogeneic SCT [11].

In a longitudinal analysis, we showed similar to other authors a relevant weight loss represented by a decrease of BMI over the course of alloSCT. Due to the intensity of the treatment, this finding is expected and has been confirmed in other studies [25, 26, 37]. The retrospective study by Rieger et al. compared patients’ weight at the time of hospital admission for alloSCT, discharge (median 41 days after alloSCT), and at the end of follow-up, which was 873 ± 361 days after transplant [37]. There was no difference between the medium BMI at these time points, providing a comparable cohort to our study with a medium follow-up of 1 year. Surprisingly, in our study, patients without a reduction in BMI after *d*_+30_ showed an increased NRM; however, this finding did not remain significant in multivariate analysis and may thus be related to other factors. For instance, other studies showed an association of GvHD with a BMI reduction [24, 26]. This was confirmed by Rieger et al., observing no meaningful difference in overall survival between patients with or without substantial weight loss. This data is contradicted by a study of Fuji et al., reporting a worse outcome (NRM and OS) of patients with a weight loss ≥ 10% after alloSCT [38].

Overall, the effect of pre-SCT BMI and its course after SCT on the outcome of SCT remains unclear. Different institutional practices of chemotherapy dose modifications according to actual or adjusted body weight as well as internal guidelines on nutritional support further reduce the comparability of the data. In addition, BMI might not represent a suitable marker for describing the nutritional status of a patient, since it does not differentiate between excess body fat, muscle mass, or surplus water. Due to the retrospective design of our study, no further analyses of patients’ body composition, e.g., by bioelectric impedance analysis, which allows an estimation of human body composition, especially body fat and muscle mass, were possible. Yet, since weight remains one of the few patient-related factors that can be influenced by reasonable interventions prior to SCT, further studies are needed to evaluate its impact.

One parameter that may represent the nutritional status of the patients more accurately is serum albumin. In our cohort, serum albumin pre-SCT as well as at 30 and 100 days after SCT was significantly associated with NRM and particularly a severe albumin deficiency (< 28 g/l) correlated strongly with a higher NRM risk. Various studies have shown that serum albumin levels both prior to SCT [39, 40] and in the post-SCT course [41, 42] correlate with outcome of SCT. Although different classifications and cut-offs for hypoalbuminemia limit the comparability of these studies, low serum albumin has been consistently associated to an increased NRM, and therefore the addition of this parameter to the HCT-CI score (“Augmented HCT-CI”) has been suggested and retrospectively validated [43, 44]. A possible explanation for this observation is the higher susceptibility towards and worse outcome of complications after allogeneic SCT in patients with hypalbuminemia, such as invasive fungal infections [45] and acute GvHD [46, 47].

Additionally, our present study for the first time to our knowledge shows that the further decrease of albumin levels in the post-SCT course is not relevant for NRM. We therefore reason that primarily albumin deficiency prior to SCT is a prognostic factor predicting increased NRM risk. This is relevant, as many other factors common in the SCT and post-SCT course influence albumin levels, e.g., hydration and sepsis [48, 49]. While low serum albumin levels are well-recognized markers of both nutritional status and liver function, it also serves as a surrogate parameter for chronic inflammation processes [50]. Among other laboratory indicators, lower albumin is associated with disease activity in patients with chronic GvHD [51]. In patients undergoing an allogeneic SCT, and therefore an intensive immunotherapeutic treatment characterized by extensive immune and inflammatory processes in the course of engraftment and further follow-up, this emphasizes the clinical relevance of pre-SCT albumin levels and underlines the difficulty of evaluating albumin levels during the course of the treatment.

In contrast to others [42], we have not found an association of serum total protein levels with NRM. In a multivariate analysis in a cohort of patients surviving 100 days after alloSCT, Wojnar et al. identified low serum total protein (< 60 g/l) on day + 100 after allogeneic SCT as an independent risk factor for NRM. The authors suggested that low serum total protein may reflect most of the complications common about 3 months after SCT, including impaired liver function as part of both acute and chronic GvHD as well as infections, malabsorption, and low immunoglobulin levels, which may additionally contribute to an elevated risk of potentially lethal infections [42]. Additionally, in a retrospective analysis by Ferreira et al., a positive correlation between serum total protein level at discharge and survival time after alloSCT was shown; yet, the influence on NRM was not analyzed [52].

In our cohort, both low serum total protein pre-SCT and on *d*_+100_ after SCT were associated with increased NRM in univariate analysis. However, this correlation was not confirmed in multivariate analysis. These findings are supported by Dietrich et al., who showed that low serum total protein (< 70 g/l) is a strong predictor of relapse in patients undergoing allogeneic SCT for AML, but has no impact on NRM [28].

Overall, data on the impact of pre-SCT serum total protein on the outcome of SCT is very limited, possibly due to its limited ability in reflecting patients’ nutritional status as it comprises many different serum proteins, including acute phase proteins and other short-lived proteins. In our cohort, only pre-SCT albumin, but not serum total protein was confirmed as a predictive marker of NRM in multivariate analysis. Yet, when assessing the nutritional status of patients, nutrition-associated blood parameters are influenced by diverse (individual) factors and are subject to their own restrictions. In the setting of allogeneic SCT especially, edema needs to be considered for BMI evaluation and stress, infections, organ dysfunctions, and gastrointestinal symptoms for evaluation of blood parameters [25, 48, 49, 53]. Therefore, nutrition-associated parameters must always be interpreted in the clinical context of the individual patient.

In sum, it is apparent that nutritional parameters and status influence the outcome after allogeneic SCT. Since the nutritional status can be modified through nutritional support, the implementation of nutritional guidelines for patients during and after allogeneic SCT should be an essential component of clinical practice. In a survey among German, Swiss, and Austrian transplant centers, all centers stated that they had established nutritional guidelines for their patients undergoing alloSCT; yet, the clinical implementation of nutritional support was very heterogenous [54]. Therefore, more data are required to facilitate the development and implementation of evidence-based nutrition guidelines for patients undergoing alloSCT.

Our present single-center cohort study was limited by its retrospective design. Moreover, its heterogeneous cohort with a relatively small number of patients limited the interpretation of the results, especially in the subgroup analyses. Given our findings, larger studies and prospective trials are needed to confirm the adverse effects of pretransplant malnutrition and determine whether improving the nutritional status prior to alloSCT by an adequate pretransplant nutritional support would lead to an improved outcome.

Our findings of serum albumin deficiency both prior to SCT as well as post-SCT being associated with NRM after allogeneic SCT may not only add to the risk evaluation and counseling of patients but may also serve as a rational for early monitoring and interventions to improve the nutritional status in at-risk patients starting prior to SCT.

## Data Availability

The datasets generated and/or analyzed during the current study are available from the corresponding author on reasonable request.
